# Neurofibromatosis to neoplasia transition: a rare case report of spindle cell malignant peripheral nerve sheath tumor with literature review

**DOI:** 10.1097/MS9.0000000000002333

**Published:** 2024-07-01

**Authors:** Adam M. Abdallah, Ramez M. Odat, Hamdah Hanifa, Zaid Shakhatreh, Qosay M. Sharqiah, Suleiman Daoud

**Affiliations:** a Department of Neurosurgery, King Abdullah University Hospital, Irbid, Jordan; b Faculty of medicine, Jordan University of Science and Technology, Irbid, Jordan; c Faculty of Medicine, University of Kalamoon, Al-Nabk, Syria; d Department of Pathology, King Abdullah University Hospital, Irbid, Jordan; e Department of Neurosurgery and Neuro-Oncology, King Abdullah University Hospital, Irbid, Jordan; f Jordan University of Science and Technology, Irbid, Jordan

**Keywords:** case report, malignant, neoplasm, neurofibromatosis, spindle cell

## Abstract

**Introduction and importance:**

Malignant peripheral nerve sheath tumor (MPNST) is a rare and aggressive soft tissue malignant tumor. MPNST in the spinal canal is rarely seen except in cases of neurofibromatosis type 1. However, a long-segment extradural spinal malignant spindle cell neoplasm has not been reported in the current literature.

**Case presentation:**

We present the first reported case of spinal malignant spindle cell neoplasm extended along the spine. The detected lesion is responsible for compressing various segments of the spinal cord, causing thinning of the cord and secondary stenosis of the spinal canal, leading to a condition known as multisegment compression myelopathy.

**Clinical discussion:**

MPNSTs are typically detected late due to nonspecific symptoms, with a higher incidence in extremities and a notable occurrence in unusual locations. Diagnosis relies on MRI and histopathology, with S_100 positivity as a neural marker. MPNSTs can arise from neurofibromas or Schwann cells, with a significant portion resulting from TP53 mutations or secondary to radiation exposure.

**Conclusion:**

This case stands out due to its unique presentation, characterized by a predominantly spindle cell morphology with certain epithelioid features. It is imperative to recognize this condition for an accurate diagnosis, emphasizing the spindle cell-type MPNST and highlighting its exceptionally poor prognosis.

## Introduction

Highlights

- Malignant peripheral nerve sheath tumor (MPNST) is a rare and aggressive tumor that requires early diagnosis and rapid management to increase the chance of survival.
- Patients with neurofibromatosis type 1 (NF1) should be monitored because it is one of the greatest risk factors for developing MPNSTs.
- MPNSTs can occur anywhere in the body. However, our case is the first reported case of spinal malignant spindle cell neoplasm extended along the spine, spanning from the C3 to L2 levels.

MPNST arise either from Schwann cells or from pluripotent cells of neural crest origin, characterized by a distinctive and severe degree of aggressiveness and malignancy^1^. In 2013, the WHO first classified MPNST as a malignant soft tissue sarcoma, accounting for approximately 5–10% of all soft tissue sarcomas^2^. A quarter to half of the diagnosed cases of this tumor coincide with or differentiate from NF1^3^. This tumor occurs in both sexes without any predilection for either, and it is relatively rare, occurring in 0.001% of individuals^4^. MRI is considered the cornerstone in the diagnosis of the tumor and in selecting the most appropriate treatment after that^5^. In this report, we describe a rare case in the medical literature of a malignant spindle cell tumor of the spine in a 55-year-old man with a past medical history of NF1. The patient was referred to the emergency department with a complaint of severe lower back pain accompanied by several complex disorders that led to his death in the end. SCARE 2023 criteria have been followed in reporting this work^6^.

## Case presentation

A 55-year-old male presented to the emergency department with severe low back pain, persisting for 4 months. Gradually worsening and radiating to both lower limbs, exacerbated by heavy exercise, relieved by simple analgesia, and more pronounced during daytime. The pain has aggravated and led to a decreased ability to walk 4 days prior to admission. Additionally, he reported reduced appetite, weight loss, and episodes of urinary retention. Physical examination showed multiple subcutaneous nodules, rubbery in consistency, and sliding under the skin but attached to underlying soft tissues, along with a micropenis. The patient’s past medical history includes diabetes mellitus, hypertension, dyslipidemia, infertility, and an ischemic stroke 4 months earlier.

**Figure 1:**
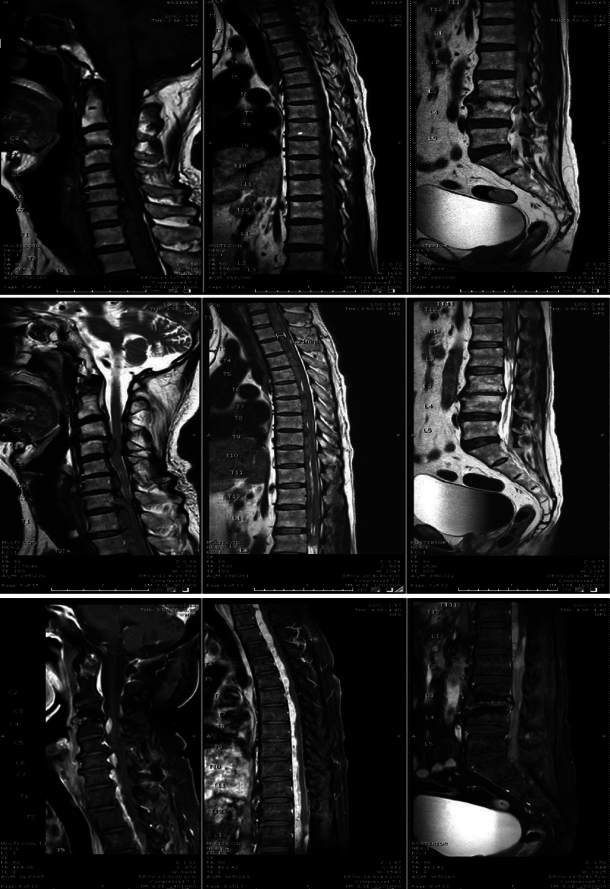
MRIs of the whole spine reveal a soft tissue lesion in the anterior – extradural space of the spine, extending from levels C3 to L2. In addition, images of the lumbar spine after gadolinium enhancement suggest L3–L4 spondylodiskitis.

Upon investigations, whole MRIs reveal the presence of a soft tissue lesion in the anterior extradural space of the spine, spanning from the C3 to L2 levels. The lesion exhibits a heterogeneous appearance, predominantly iso-intense on both T1 and T2 images, and demonstrates vivid enhancement on postcontrast images. It contains multiple foci of low T1/T2 intensities, primarily indicative of calcifications. Postgadolinium lumbar MRI suggestive of L3–L4 spondylodiskitis (Fig. 1) Furthermore, there are notable extraspinal findings, including bilateral renal lipomatosis and renal cortical cysts. Additionally, a small tracheal polyp is observed at the T2 level.

**Figure 2:**
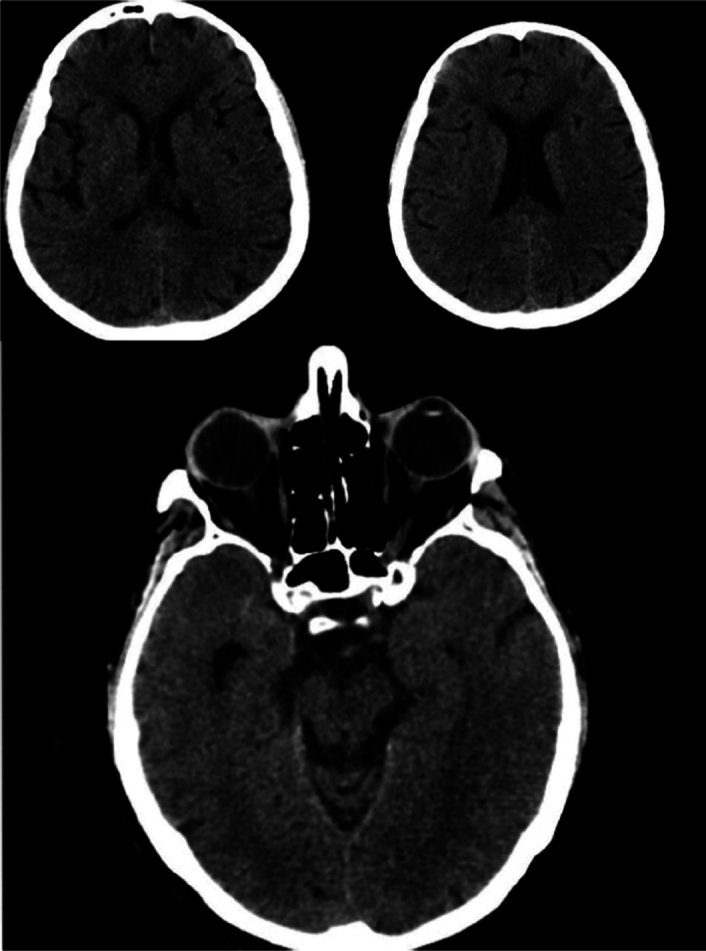
The scan revealed low-attenuation foci in the left posterior centrum semiovale, consistent with a known acute/subacute infarct history. Multiple low-attenuation areas in both cerebral hemispheres indicated past ischemic events. An old lacunar infarct was noted in the pons, along with periventricular deep white matter ischemic changes.

On the hospital day, an urgent brain computed tomography (CT) scan was done due to a sudden decrease in the patient’s sensorium. The pathological results revealed several hypodense foci in both cerebral hemispheres, consistent with previous infarctions experienced by the patient (Fig. 2). The cerebellum appeared within normal limits, and no signs suggestive of hemorrhage or intracranial masses were observed.

**Figure 3:**
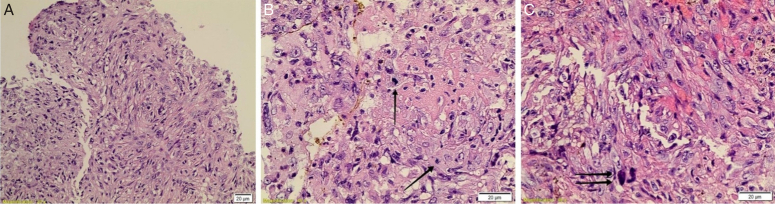
(A) Current tumor showing pleomorphic spindle cells arranged in fascicles and bundles (×200 total magnification). (B) High power view (×400) showing occasional mitotic figures (arrows) and necrotic area mainly on the right side of the picture. (C) High power view (×400) of the tumor with occasional bizarre cells (double arrows).

**Figure 4:**
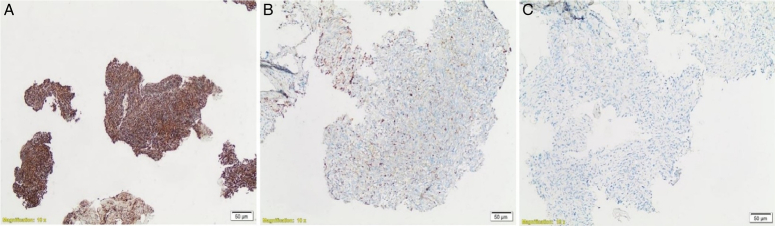
(A) Vimentin immunohistochemical (IHC) stain (diffuse strong positivity in tumor cells). (B) Keratin AE1/AE3 IHC stain (scattered positive cells). (C) S100 IHC stain (negative in tumor cells).

**Figure 5:**
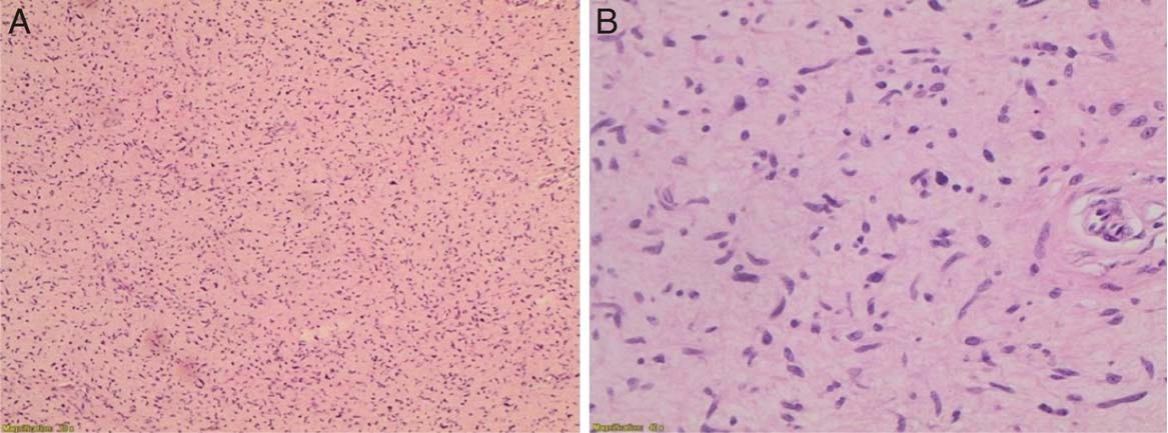
(A) The previous biopsy (×100) showed a spindle cell lesion. (B) High power view (×400) showing spindle cells with serpentine-shaped nuclei and occasional atypical cells embedded in myxoid stroma.

**Figure 6:**
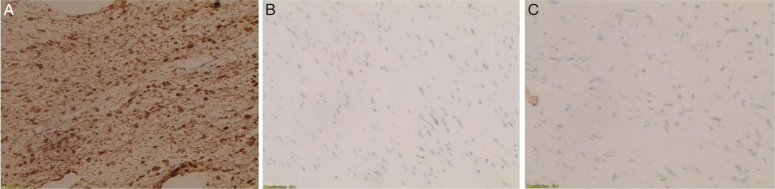
(A) S100 immunohistochemical (IHC) stain (×40) showing diffuse nuclear and cytoplasmic staining in tumor cells. (B) SMA IHC stain (negative in tumor cells). (C) Desmin stain (negative in tumor cells).

**Figure 7:**
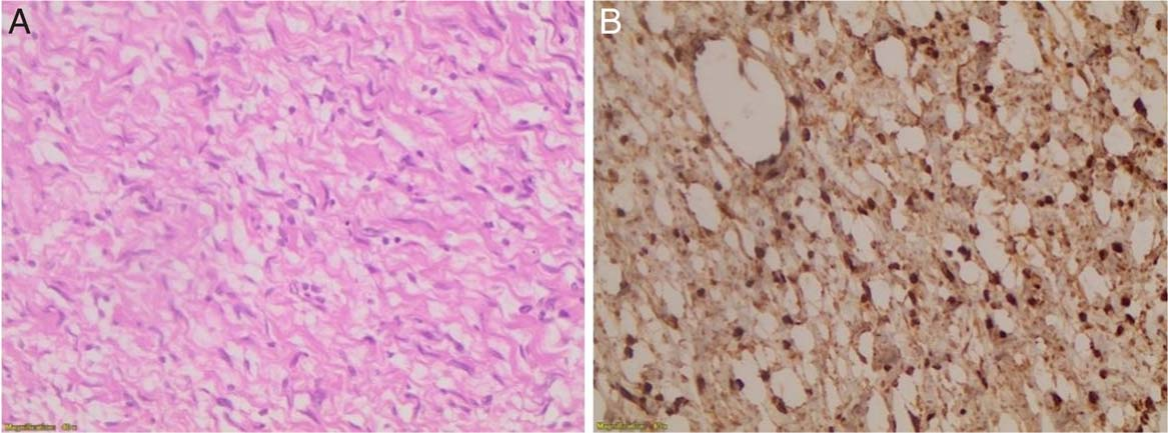
(A) High power view (×400) showing spindle cell tumor with serpentine-shaped nuclei. (B) S100 immunohistochemical (IHC) stain showing diffuse nuclear and cytoplasmic staining in tumor cells.

To improve the quality of life, the patient underwent an L1–L2 decompressive laminectomy for tumor debulking and to obtain a soft tissue excisional biopsy of the spinal cord lesion. However, there was no improvement in his symptoms postoperatively. He also complained of severe coccydynia, leading to a consultation with a pain management specialist. The specialist recommended ganglion impar block and bilateral L5–S1 facet joint steroid injections under CT guidance. At the same time, an interventional radiologist was asked to perform a CT-guided bone biopsy from the L3 vertebral body to search for spondylodiskitis. The histopathologic examination of the spinal cord lesion revealed a malignant mesenchymal tumor composed of atypical and pleomorphic spindle cells. The tumor cells had oval hyperchromatic nuclei, conspicuous nucleoli, and eosinophilic cytoplasm. Focal necrosis, occasional bizarre cells, and mitotic figures were identified (Fig. 3). Immunohistochemistry showed positive results for vimentin and only focal positivity for keratin cocktail AE1/AE3. The tumor was negative for other markers such as S100, SOX10, CD34, ERG, SMA, desmin, melan-A, HMB-45, EMA, progesterone, and GFAP (Fig. 4). INI-1, H3K27me3, and BRG-1 were retained in tumor cells, confirming the diagnosis of MPNST. The CT-guided bone biopsy confirmed tuberculosis spondylodiskitis using TB PCR serology testing. In addition, a biopsy of a right shoulder skin lesion revealed a neurofibroma with atypia. The histopathologic examination showed a dermal ill-defined spindle cell tumor with occasional atypical pleomorphic cells in a background of myxoid stroma (Fig. 5). Immunohistochemistry showed diffuse positivity for S100 and negativity for SMA and desmin (Fig. 6). Moreover, there was an old history of a diagnosis of neurofibroma in a wrist mass before 13 years, where the tumor cells were strongly and nearly diffusely immunoreactive for S100 (Fig. 7).

After surgery, the patient received intensive rehabilitation and pain management. However, his mobility remained significantly impaired, requiring the use of a wheelchair. The patient was also on anticoagulants due to a high risk of thromboembolism. Despite follow-up for 2 years, the patient developed a pulmonary embolism due to immobility and hypercoagulability, leading to his death.

## Discussion

### Epidemiology

MPNSTs, also known by various names such as neurofibrosarcoma and malignant schwannoma, are rare (0.001%) and aggressive tumors. Their aggressive behavior and poor prognosis stem from the fact that they are often detected in their advanced stages due to their slow growth and initially nonspecific symptoms, which may include pain and neurological deficit^7^. MPNSTs ranks sixth in terms of the prevalence of soft tissue sarcomas, with a rate of 5–10% of them. It can also be developed either from pre-existing neurofibroma or through Schwann cells or when the tumor develops in the peripheral nerves themselves, specifically the peripheral nerves in the thigh and buttocks, such as the sciatic nerve^8,9^. It typically occurs between the second and fifth decades of life, with an average age of 35 years, and has a 10–20% occurrence rate in children^10,11^.

### Causes and symptoms

There is a close relationship between MPNST progression and the presence of TP53 mutations or schwannoma, especially when there is a previous history of NF1^12^. Most cases of NF1 are benign, but it is one of the most common tumors that can transform into malignancies, including MPNST, and occurs in approximately one in every 2500 individuals worldwide^13^. Although 10% of NF1 progresses to MPNST and accounts for approximately half of its cases, neurofibromatosis type 2 should also be considered during diagnosis despite its rarity in association with MPNST^14^. The other half of MPNST cases result from different mutations (45%) or occur as secondary malignancies following radiation exposure (5%)^15^. Initially, painless soft tissue masses are common symptoms, gradually progressing to cause pain and sensory disturbances^16^. In our case, the patient had a previously diagnosed neurofibroma in the wrist 13 years prior and later experienced severe lower back pain that worsened over several months, rendering him unable to walk, raising suspicions of MPNST. Gnanalingham *et al.* ^17^ reported an MPNST case in the thoracic spine of a woman who presented with mid-back pain and hemiparesis with a sensory deficit at the T4 level. Arnesen and Jones^18^ described symptoms resulting from spinal cord compression due to spindle cell sarcoma in a patient with a history of uterine neurofibroma. To our knowledge, no previous cases of spindle cell-type MPNST involving most spinal levels (C3–L2) have been reported as in our case. Previously published papers regarding MPNSTs in the spine were mostly concerned either with the thoracic spine only or, as reported by Suzuki *et al.* ^19^ where the tumor was at the lumbar spine level.

### Reported cases

Studies have indicated that MPNSTs are more common in the extremities, followed by the trunk, head, and neck^20^. However, there have been extremely rare cases of MPNSTs occurring in unusual locations, such as the abdominal wall in a patient with Lynch syndrome^21^. The MPNST of the trigeminal nerve was reported by Liang *et al.* ^8^ in an elderly patient, as well as in the vagus nerve in another study^3^. In addition, a number of cases have been documented regarding the possibility of it occurring in the lungs^7,22^. Notably, the first and youngest case of MPNST in the colon was reported in a 2-day-old infant, and in 2017, the first case of MPNST in the lower eyelid was documented^23,24^ (Table 1).

**Table 1:** Reports of malignant peripheral nerve sheath tumor in various locations of the body.

Case description	References	Location of tumor	Patient demographics	Presentation	Diagnostic methods	Treatment
Case 1	Hasnaoui *et al.* ^21^	Abdominal wall	39-year-old male	Personal history of colonic cancer: then the patient presented with an asymptomatic lump in the abdominal wall	CT imaging and histopathological examination	Surgery
Case 2	Liang *et al.* ^8^	Trigeminal nerve	56-year-old female	2-month-long history of numbness of the right face and progressive weakness of the left limbs	CT, histopathologic, and immunochemical examinations	Surgery and adjuvant radiotherapy
Case 3	Borovika and Isajevs^3^	Vagus nerve	62-year-old female	Right-sided neck mass over the past 3 months that gradually increased in size	CT, MRI, histopathologic examination, and immunohistochemistry	Surgery and adjuvant radiotherapy
Case 4	Abdallaoui *et al.* ^7^	Lungs	68-year-old male	Twelve-day history of visual disturbances, confusion, and headaches	CT, MRI, liver biopsy, histopathologic examination, and immunohistochemical analysis	Biologic agent (Imatinib) and radiotherapy
Case 5	Lee *et al.* ^23^	Colon	2-day-old female neonate	Poor oral feeding and bilious vomiting	CT, histopathologic examination, and immunohistochemical analysis	Surgery
Case 6	Lindsay *et al.* ^24^	Lower eyelid	55-year-old male	Multiple recurrent lower eyelid masses	Histopathologic examination and immunohistochemical analysis	Surgery and adjuvant radiotherapy

### Diagnostics

Early diagnosis is crucial despite the challenges faced by physicians during the diagnostic process. The MRI is the most accurate means for diagnosing MPNST, while fine-needle aspiration is preferable in cases suspected of recurrence or metastasis^5^. Chemically, positive S_100 is considered a distinctive and specific neural marker in MPNST tumors, which aligns with our case. However, definitive diagnosis requires histopathological examination to identify characteristic features, such as asymmetric spindle cells, as observed in our patient.

### Possible treatment

Regarding the treatment of MPNST, several studies have shown the limited importance of radiation therapy. Additionally, chemotherapy has proven insufficient, and its use has even exacerbated outcomes in patients who underwent it^25^. In contrast, complete surgical excision with clear margins has been highly effective in treatment. Unfortunately, even with surgery, the tumor recurrence rate remains very high, leading to elevated mortality rates within 5 years of diagnosis and treatment^26^.

### Prognosis

Certain features, including tumor size greater than 5 cm, irregular margins, deep location with surrounding edema, and distant metastases, indicate a very poor prognosis^27^. The mortality rate associated with MPNST occurring in the head and neck is 66.7%, while in the trunk, it is 48.8%, and in the extremities, it averages 27.5%, and the average duration of a patient’s survival is 32 months^28,29^. In our case, multiple complications rapidly developed in the patient, compounded by a complex medical history that included conditions such as stroke, tuberculosis, pulmonary issues, and NF1. Despite the doctors’ efforts to perform necessary interventions to save the patient’s life and prolong survival, the patient ultimately passed away.

## Conclusion

MPNSTs are among the most aggressive and malignant tumors. Therefore, in this case, we emphasize the importance of monitoring patients with neurofibromatosis and conducting necessary investigations when suspecting MPNST development, given their strong association with each other, to improve survival rates as much as possible.

## Ethical approval

Not applicable.

## Consent

Written informed consent was obtained from the patient’s family for the publication of this case report and accompanying images. A copy of the written consent form is available for review by the editor-in-chief of this journal upon request.

## Source of funding

Not applicable.

## Author contribution

A.M.A.: validation, visualization, writing – original draft, and writing – review and editing; R.M.O: validation, visualization, writing – original draft, and writing – review and editing; H.H.: writing – original draft and writing – review and editing; Z.S.: writing – original draft and writing – review and editing; Q.M.S.: resources, writing – original draft, and writing – review and editing; S.D.: conceptualization, visualization, and writing – review and editing.

## Conflicts of interest disclosure

The authors declare that they have no competing interest.

## Research registration unique identifying number (UIN)

Not applicable.

## Guarantor

Dr Adam Abdallah.

## Data availability statement

There is no data analyzed or produced in this research.

## Provenance and peer review

I agree.
